# A Standardized Small Sided Game Can Be Used to Monitor Neuromuscular Fatigue in Professional A-League Football Players

**DOI:** 10.3389/fphys.2018.01011

**Published:** 2018-08-07

**Authors:** Amber E. Rowell, Robert J. Aughey, Jo Clubb, Stuart J. Cormack

**Affiliations:** ^1^Institute of Health and Sport, Victoria University, Melbourne, VIC, Australia; ^2^Seattle Sounders Football Club, Seattle, WA, United States; ^3^Buffalo Bills NFL, Buffalo, NY, United States; ^4^Buffalo Sabres NHL, Buffalo, NY, United States; ^5^School of Exercise Science, Australian Catholic University, Melbourne, VIC, Australia

**Keywords:** countermovement jump, soccer, training load, GPS, accelerometer, PlayerLoad^TM^

## Abstract

**Introduction:** Training and competition load can cause neuromuscular fatigue (NMF) and modified movement strategy such as an increase in the contribution of the medio-lateral [PlayerLoad^TM^_ML_(%)] and decrease in the % vertical [PlayerLoad^TM^_V_(%)] vectors, to total PlayerLoad^TM^ (accelerometer derived measurement in vertical, medio-lateral, and anterior-posterior planes) in matches. NMF assessment involves expensive equipment, however, given the modification of match movement strategy with NMF, this may be present in a standardized drill. The aim of this study was to determine the utility of a small sided game (SSG) for the measurement of NMF.

**Materials and Methods:** Data was collected throughout a competitive football season. External load was quantified using global positioning system (GPS) and accelerometry, and internal load by session rating of perceived exertion (sRPE). A 5 vs. 5 SSG and countermovement jump (CMJ), for determination of flight time:contraction time (FT:CT), were performed the day prior to each match. Weekly volume from GPS, PlayerLoad^TM^ and sRPE were calculated across the season. Weekly SSG activity profile and FT:CT was compared between “high” and “low” load weeks determined relative to season average. SSG activity profile was assessed between weeks where FT:CT was above or below pre-season baseline. Impact on match activity profile was examined between weeks where FT:CT and SSG activity profile were higher or lower than baseline. The difference (high vs. low load and < or > pre-season baseline) was calculated using the effect size (ES) ± 90% CI and practically important if there was a >75% likelihood of exceeding an ES of 0.2.

**Results:** All weekly load metrics increased SSG PlayerLoad^TM^⋅m⋅min^-1^ when above season average, however, the impact on FT:CT was trivial. Reduced weekly FT:CT compared to baseline resulted in lower SSG PlayerLoad^TM^⋅min^-1^ and PlayerLoad^TM^Slow⋅min^-1^. FT:CT below baseline increased match PlayerLoad^TM^_ML_(%) and decreased PlayerLoad^TM^_V_(%) during subsequent match play. Similarly, a reduction in SSG PlayerLoad^TM^⋅m⋅min^-1^ was followed by increased match PlayerLoad^TM^_ML_(%).

**Conclusion:** Changes in select match activity profile variables following a reduction in SSG PlayerLoad^TM^ m.min^-1^, mirror those seen when FT:CT is reduced. Increased PlayerLoad^TM^_ML_(%) during matches likely represents fatigue driven modification to movement strategy. Small-sided games may be a useful tool to detect NMF.

## Introduction

Monitoring an athlete’s response to training and competition load is important for improving adaptation and reducing the risk of illness and injury (Halson, 2014). Quantification of training and competition load can include measures of internal [e.g., heart rate, rating of perceived exertion (RPE)] and external load (e.g., distance, number of repetitions) (Buchheit, 2014). The availability of microtechnology devices such as global positioning system (GPS) has made the collection of external load variables such as speed and distance commonplace (Aughey, 2011). However, the data relating to short, high speed efforts has validity and reliability limitations as speed and movement complexity increases (Jennings et al., 2010). In addition, speed and distance metrics fail to account for movements associated with team sports such as changing direction and jumping (Cormack et al., 2013; Barrett et al., 2015). To overcome this, tri-axial accelerometers sampling at 100 Hz have been increasingly used as measures of external load in team sports, with the most commonly utilized metric referred to as PlayerLoad^TM^ (Boyd et al., 2013).

PlayerLoad^TM^, calculated as the sum of instantaneous rate of change from the individual vertical (PlayerLoad^TM^_V_), medio-lateral (PlayerLoad^TM^_ML_) and anterior-posterior (PlayerLoad^TM^_AP_) planes, has high validity and reliability (Cormack et al., 2013; Barrett et al., 2014). Match activity profiles in football have been quantified using PlayerLoad^TM^ (Rowell et al., 2016), and the impact of neuromuscular fatigue (NMF) on individual PlayerLoad^TM^ vectors has also been assessed in Australian Rules Football (Cormack et al., 2013; Mooney et al., 2013). Furthermore, changes in the contribution of individual vectors to PlayerLoad^TM^ have been studied in both soccer match simulation as well as professional match play (Barrett et al., 2015, 2016b).

In addition to between-match changes in activity profile, the subsequent physical and psycho-physiological response of athletes to match play has also been reported (Cormack et al., 2013; Gescheit et al., 2015; Rowell et al., 2016). Numerous variables have been measured in high performance sport for this purpose (e.g., NMF via jump testing and hormonal concentration via saliva); however, they require additional time for testing and the use of specialized equipment (Taylor et al., 2012; Cormack et al., 2013; Mooney et al., 2013). As changes to the contribution of individual PlayerLoad^TM^ vectors occur when matches are played in the presence of NMF, it could be predicted that these changes are also evident in training drills (Cormack et al., 2013; Barrett et al., 2015; Gescheit et al., 2015). In fact, standardized field protocols are commonly used for the assessment of autonomic nervous system status (Buchheit et al., 2008, 2010, 2012). Given the demonstrated reliability of numerous activity profile metrics during small sided games (SSGs), there may be potential for a standardized version of such a drill to be used as a tool to assess neuromuscular function (Stevens et al., 2016). However, the impact of previous training and competition load on SSG activity profile has not been examined. Critically, the impact of NMF on subsequent match performance has received little attention (Andersson et al., 2008; Cormack et al., 2008b; Mooney et al., 2011).

Increased weekly training load greater than 15% from the previous week is associated with an increased injury risk (Gabbett, 2016). Whilst it has been suggested that a relatively high acute load increases injury risk, the precise mechanism by which this occurs is unclear. It is possible that relatively high acute load manifests as a modified movement strategy, therefore placing tissue at increased risk of injury (Oliver et al., 2014). Conversely, enhanced match performance in Australian Rules Football was linked to a higher acute load, and training stress balance, than losses (Aughey et al., 2016). Indeed, the acute training load is said to represent “fatigue” in Banister’s model and may drive adaptation (Banister et al., 1975; Aughey et al., 2016). These findings suggest there may be a trade-off between injury risk and adaptation/performance; therefore, tracking responses to acute load and potential movement strategy alterations are of particular interest.

Improving the training process is a critical challenge for coaching and sport science support staff in professional football. The ability to assess changes in movement strategy that provides insight into the fatigue status of athletes via a common training drill is an attractive proposition. Therefore, the purpose of this study was to assess:

(1) The impact of preceding weekly load on SSG activity profile and matchday -1 (the day prior to the match) NMF.
(2) The degree to which a change in matchday -1 NMF impacts SSG activity profile.
(3) The impact of matchday -1 NMF and SSG activity profile on subsequent match activity profile.

## Materials and Methods

Data was collected from 21 male outfield football players competing in the elite Australian football competition – the A-League. Players had a mean ± SD age; 25.2 ± 5.5 years, height; 180 ± 6.7 cm and mass; 75.6 ± 5.9 kg. Data was collected from a single competitive season and therefore provides a case study of this particular club. Ethical approval was granted from Victoria University Human Research Ethics Committee, with written informed consent obtained prior to commencement.

### Training Structure

One match on average was played per week with 3–4 main training sessions preceding the match. Data included in the analysis was collected from a total number of 110 training sessions, and 36 matches across the competition season. Training load reflects pitch-based skills sessions only; given the weekly (on average < 1 h) gym based session was targeted toward injury prevention exercises. The competitive 2015/2016 A-league season spanned over 7 months (October–April). During the latter part of the season (February–May), the team also competed in the Asian Champions League (ACL). The ACL cross-over period resulted in multiple matches played per week and reduced training sessions. There were 7 weeks across the season where the team played two matches per week due to a pre-season FFA cup, a game re-scheduling and the ACL period. The overall training design and implementation was under the control of coaching and fitness staff and was not modified for this study.

### External and Internal Load

During each match and training session, athletes wore a global positioning system (GPS) device (OptimEye S5; Catapult Sports, Melbourne, VIC, Australia) sampling at 10 Hz, which also housed accelerometers sampling at 100 Hz (Kionix: KXP94). The unit was worn in a custom tight fitting vest, with athletes assigned the same unit throughout the season. External load variables selected to reflect total weekly volume were: distance (m), high-intensity running (HIR) distance (m > 4.2 m.s^-1^) and PlayerLoad^TM^ (au). Following each training session (≈30 min post) athletes recorded their RPE, which was multiplied by the duration of the session or playing time in the match to give session RPE (sRPE) as a measure of internal load (Foster, 1998).

### Small-Sided Game

A standardized small-sided game (SSG) drill: 5v5 + 5 + goal keepers (GKs); two teams of five outfield players with a GK each, and the third team of five acting as “bouncers” on the outside of the pitch to keep the ball in play, was performed. The SSG drill was played within a 45 m × 36 m area of the outdoor training pitch (natural turf), and was a free-play style with no restrictions, with the outcome aim to score as often as possible. Once the teams had been assigned by coaching staffs they remained across the rotation and each team played the others. The SSG was performed after the warm up, at the same point of the weekly cycle; the morning before the training session 1 day prior to the match (matchday -1) which was at least 42 h after the previous match. The SSG teams were selected by coaching staff. A total number of four sets performed, with each team completing two sets of 3 min with 1 min of rest between each set. On average, players participated in 85% of SSG time throughout the season.

### SSG and Match Activity Profile

The following variables (per minute of activity time) were collected from the match and SSG: meters per minute (m⋅min^-1^), PlayerLoad^TM^ per minute (PlayerLoad^TM^⋅min^-1^), PlayerLoad^TM^ per meter per minute (PlayerLoad^TM^⋅m⋅min^-1^), PlayerLoad^TM^ Slow per minute (movement in all three planes when velocity was < 2 m⋅s^-1^; PlayerLoad^TM^Slow⋅min^-1^), PlayerLoad^TM^2D per minute (all movements performed excluding the vertical vector; PlayerLoad^TM^2D⋅min^-1^), and the percent contributions of individual PlayerLoad^TM^ vectors [PlayerLoad^TM^_AP_(%), PlayerLoad^TM^_ML_(%) and PlayerLoad^TM^_V_(%)]. Only players who played greater than 75% of match time were included in the analysis.

### Matchday -1 Neuromuscular Fatigue

Prior to the training session that contained the SSG drill, athletes performed a maximal countermovement jump (CMJ) on a force plate (400 Series Platform Plate; Fitness Technology, Adelaide, SA, Australia) connected to manufacturer-supplied software (Ballistic Measurement System; Fitness Technology, Adelaide, SA, Australia). Athletes were familiarized with the CMJ during the pre-season and in line with established protocols, were instructed to jump as high as possible (Cormack et al., 2008a). The flight time:contraction time (FT:CT) ratio is the measure most sensitive to alterations in training and competition load in this cohort, and was therefore used to determine athletes pre-match NMF status (Cormack et al., 2008c; Rowell et al., 2016).

### Weekly Load

To identify the impact of weekly load on SSG and FT:CT they were both performed at the same time during the training microcycle; matchday -1. “Weekly” load was therefore calculated from the preceding matchday -1 session through to the day prior to the SSG training session of the next week. This included one game per “training week.” For each athlete, their individual average weekly volume [total distance, HIR distance, total PlayerLoad^TM^ and internal load (sRPE)] was calculated across the season. A “high” weekly load was then calculated as any week when the individual athletes’ load (for each variable) was above their individualized seasonal average and “low” when their weekly load metric was below their season average for each individual variable.

### Reliability and Baseline Calculation

**FIGURE 1:**
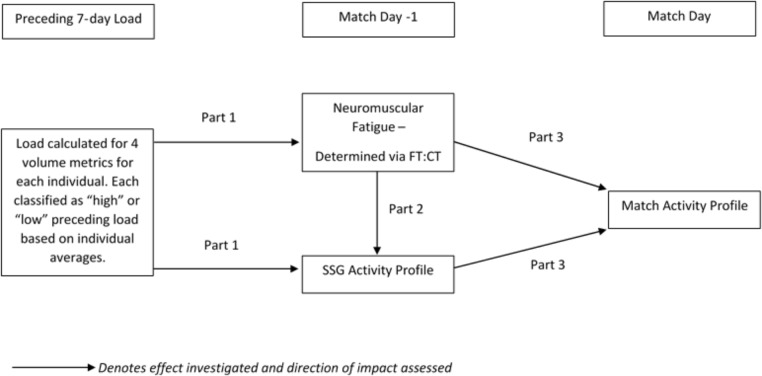
Diagrammatic representation of the steps of analysis. Small-sided game; SSG, Flight time:Contraction time ratio; FT:CT.

The reliability of activity profile variables, represented by the coefficient of variation (CV%) (Hopkins, 2017b), was calculated during five consecutive pre-season weeks where the SSG was performed on a matchday -1. Based on previous work by us, the CV% of FT:CT was set at 8% (Cormack et al., 2008c). The average of athletes pre-season trials was also used to calculate their baseline SSG activity profile variables and FT:CT for later comparison. **Figure 1** shows a diagrammatic representation of the analysis process described in further detail below.

Data were log-transformed to reduce bias due to non-uniformity of error. The magnitude of the mean difference (high vs. low load and < or > baseline) was calculated using the ES ± 90% CI. Effects were classified as small; 0.20–0.60, moderate; 0.60–1.20, large 1.2–2.0, very large; 2.0–4.0 and extremely large; >4.0. Differences were declared practically important if there was a >75% likelihood of exceeding the smallest important ES (0.2) threshold (Hopkins, 2004). Differences with less certainty were considered trivial, and where the 90% CI crossed substantially positive and negative values the effect was considered ‘unclear’ (Hopkins, 2004).

Part 1 – Impact of weekly load on SGG activity profile and NMF

Step 1: For each individual athlete, weekly in-season SSG activity profile (using variables deemed reliable from above) and FT:CT was compared between “high” weekly load (those weeks above the season average) and “low” weekly load (those weeks below season average) using a custom spreadsheet (Hopkins, 2017a). The SSG activity profile metrics that were >75% likely to be different when comparing “high” and “low” load weeks were retained for further analysis (Step 4).

Part 2 – Impact of difference in NMF (FT:CT) on SSG activity profile

Step 2: The difference in SSG activity profile was compared between weeks where an athlete’s FT:CT was above or below their pre-season baseline by >8% CV (FT:CT lower than baseline by >8.0% was considered “fatigued”).

Part 3 – Impact of difference in FT:CT and SSG activity profile on match activity profile

Step 3: The impact on match activity profile was compared between weeks where FT:CT was higher or lower than pre-season baseline (average of 5 pre-season measurements) by more than the 8% CV.

Step 4: In order to compare the impact of differences in SSG activity profile metrics on match exercise intensity using the same approach as was taken for FT:CT (Step 3 above) the difference in match exercise intensity was compared between weeks where the SSG activity profile metrics (retained from Step 1 above) were higher or lower than pre-season baseline by more than the 1.0, 1.5, and 2.0x %CV for that SSG metric (calculated from SSG reliability trials).

## Results

### SSG Reliability Analysis

The CV% values from the reliability analysis of the SSG were: m⋅min^-1^: 4.2%, HIR (>4.2 m⋅s^-1^)⋅min^-1^: 30.6%, PlayerLoad^TM^⋅min^-1^: 4.5%, PlayerLoad^TM^⋅m⋅min^-1^: 2.8%, PlayerLoad^TM^2D⋅min^-1^: 4.6%, PlayerLoad^TM^Slow⋅min^-1^: 8.9%, PlayerLoad^TM^_AP_(%): 3.9%, PlayerLoad^TM^_ML_(%): 2.4%, PlayerLoad^TM^_V_(%): 2.1%. These variables were considered reliable and were used for subsequent analysis, however, due to a poor CV of 30.6% (Jennings et al., 2010), HIR.min^-1^ was excluded from further analysis.

Based on the response to high load (i.e., >75% likely to be different between weeks above and below season average), the values retained for analysis from the SSG were PlayerLoad^TM^ PlayerLoad^TM^ m⋅min^-1^ and PlayerLoad^TM^Slow⋅min^-1^.

### Impact of Weekly Load on SSG Activity Profile and FT:CT

The average weekly “high” and “low” load values for distance, HIR, PlayerLoad^TM^ and internal load are presented in **Table 1**. The values displayed in **Table 1** are the team average of each individual athlete’s “high” and “low” weeks which was calculated relative to their individualized season average.

**Table 1:** Average weekly total distance, high-intensity running (HIR; >4.2 m⋅s^-1^), PlayerLoad^TM^ and internal load reflecting “high” load weeks compared to “low” load weeks.

	Total distance (m) (mean ± SD)	HIR (m; >4.2 m⋅s^-1^) (mean ± SD)	PlayerLoad^TM^ (au) (mean ± SD)	Internal load (au) (mean ± SD)
Team average “high load” > season average	22820 ± 2687 m	3047 ± 574 m	2402 ± 365 au	1930 ± 58 au
Team average “low load” < season average	8132 ± 2676 m	841 ± 291 m	856 ± 299 au	728 ± 302 au

The mean ± SD and change (ES ± CI) in SSG activity profile metrics and FT:CT when weekly load was above and below the season average is displayed in **Table 2**. There were a number of substantial changes (small-moderate effects) in SSG metrics as a result of high weekly load. There was a *very likely* increase in PlayerLoad^TM^⋅m⋅min^-1^ when all weekly load metrics were above the season average. Similarly, a *very likely* increase in SSG PLSlow⋅min^-1^ with a higher weekly total distance, PlayerLoad^TM^ and internal load, and *likely* increase from a higher weekly HIR compared to season average. There was also a *likely* increase in PlayerLoad^TM^2D⋅min^-1^ when weekly total distance, HIR and PlayerLoad^TM^ were above the season average. “High” weekly load metrics caused trivial changes in FT:CT.

**Table 2:** Mean ± SD and change in mean (ES ± 90% CI) in selected small-sided game; SSG activity metrics when weekly load variable was higher than the season average.

Weekly load metric	SSG Variable and FT:CT	Mean ± SD when weekly load metric < Season Average “Low Week”	Mean ± SD when weekly load metric > Season Average “High Week”	ES ± 90%CI change in SSG variable
**Total distance**	m⋅min^-1^	126.3 ± 10.2	125.4 ± 11.0	-0.09 @ 0.37
	PlayerLoad^TM^⋅min^-1^	14.1 ± 1.4	14.6 ± 1.5	0.33 @ 0.28**
	PlayerLoad^TM^⋅m⋅min^-1^	0.112 ± 0.008	0.117 ± 0.008	0.61 @ 0.19***
	PlayerLoad^TM^2D⋅min^-1^	8.58 ± 0.86	8.92 ± 0.94	0.38 @ 0.23**
	PlayerLoad^TM^Slow⋅min^-1^	4.23 ± 0.53	4.58 ± 0.52	0.60 @ 0.32***
	PlayerLoad^TM^_AP_(%)	26.5 ± 2.0	26.4 ± 1.5	-0.04 @ 0.27
	PlayerLoad^TM^_ML_(%)	26.0 ± 2.0	26.3 ± 1.6	0.18 @ 0.15
	PlayerLoad^TM^_V_(%)	47.5 ± 2.0	47.3 ± 2.0	-0.11 @ 0.19
	FT:CT	0.68 ± 0.12	0.68 ± 0.11	-0.01 @ 0.13
**HIR distance (>4.2 m⋅s**^-^**^1^)**	m⋅min^-1^	127.0 ± 8.5	124.8 ± 10.9	-0.27 @ 0.40
	PlayerLoad^TM^⋅min^-1^	14.3 ± 1.2	14.6 ± 1.5	0.25 @ 0.27
	PlayerLoad^TM^⋅m⋅min^-1^	0.113 ± 0.008	0.118 ± 0.008	0.57 @ 0.20***
	PlayerLoad^TM^2D⋅min^-1^	8.66 ± 0.78	8.92 ± 0.94	0.30 @ 0.24**
	PlayerLoad^TM^Slow⋅min^-1^	4.30 ± 0.48	4.58 ± 0.56	0.55 @ 0.41**
	PlayerLoad^TM^_AP_(%)	26.5 ± 2.0	26.4 ± 1.5	-0.05 @ 0.27
	PlayerLoad^TM^_ML_(%)	26.1 ± 2.0	26.3 ± 1.6	0.08 @ 0.14
	PlayerLoad^TM^_V_(%)	47.4 ± 2.0	47.3 ± 2.0	-0.09 @ 0.18
	FT:CT	0.68 ± 0.11	0.68 ± 0.11	-0.02 @ 0.13
**PlayerLoad^TM^**	m⋅min^-1^	126.1 ± 9.9	125.4 ± 11.3	-0.08 @ 0.39
	PlayerLoad^TM^⋅min^-1^	14.1 ± 1.4	14.6 ± 1.5	0.35 @ 0.28**
	PlayerLoad^TM^⋅m⋅min^-1^	0.112 ± 0.008	0.117 ± 0.007	0.58 @ 0.19***
	PlayerLoad^TM^2D⋅min^-1^	8.57 ± 0.87	8.92 ± 0.94	0.38 @ 0.23**
	PlayerLoad^TM^Slow⋅min^-1^	4.24 ± 0.55	4.58 ± 0.52	0.58 @ 0.33***
	PlayerLoad^TM^_AP_(%)	26.5 ± 2.0	26.4 ± 1.5	-0.01 @ 0.27
	PlayerLoad^TM^_ML_(%)	26.9 ± 1.7	26.5 ± 1.6	-0.23 @ 0.23
	PlayerLoad^TM^_V_(%)	47.5 ± 2.0	47.3 ± 2.0	-0.09 @ 0.19
	FT:CT	0.68 ± 0.12	0.68 ± 0.11	-0.01 @ 0.13
**Internal load**	m⋅min^-1^	126.5 ± 10.0	125.4 ± 10.8	-0.11 @ 0.35
	PlayerLoad^TM^⋅min^-1^	14.2 ± 1.4	14.6 ± 1.5	0.23 @ 0.28
	PlayerLoad^TM^⋅m⋅min^-1^	0.113 ± 0.008	0.117 ± 0.007	0.44 @ 0.14***
	PlayerLoad^TM^2D⋅min^-1^	8.68 ± 0.90	8.87 ± 0.94	0.20 @ 0.25
	PlayerLoad^TM^Slow⋅min^-1^	4.26 ± 0.56	4.52 ± 0.45	0.46 @ 0.26***
	PlayerLoad^TM^_AP_(%)	26.7 ± 1.6	26.4 ± 1.6	-0.19 @ 0.24
	PlayerLoad^TM^_ML_(%)	26.0 ± 1.9	26.3 ± 1.6	0.15 @ 0.15
	PlayerLoad^TM^_V_(%)	47.3 ± 2.0	47.3 ± 2.0	0.02 @ 0.14
	FT:CT	0.68 ± 0.12	0.68 ± 0.11	0.04 @ 0.11


Symbols denote a ^∗∗^likely, ^∗∗∗^very likely, and ^∗∗∗∗^most likely chance of the effect size; ES exceeding the smallest important value of 0.2.

### Impact of FT:CT on SSG Activity Profile

**FIGURE 2:**
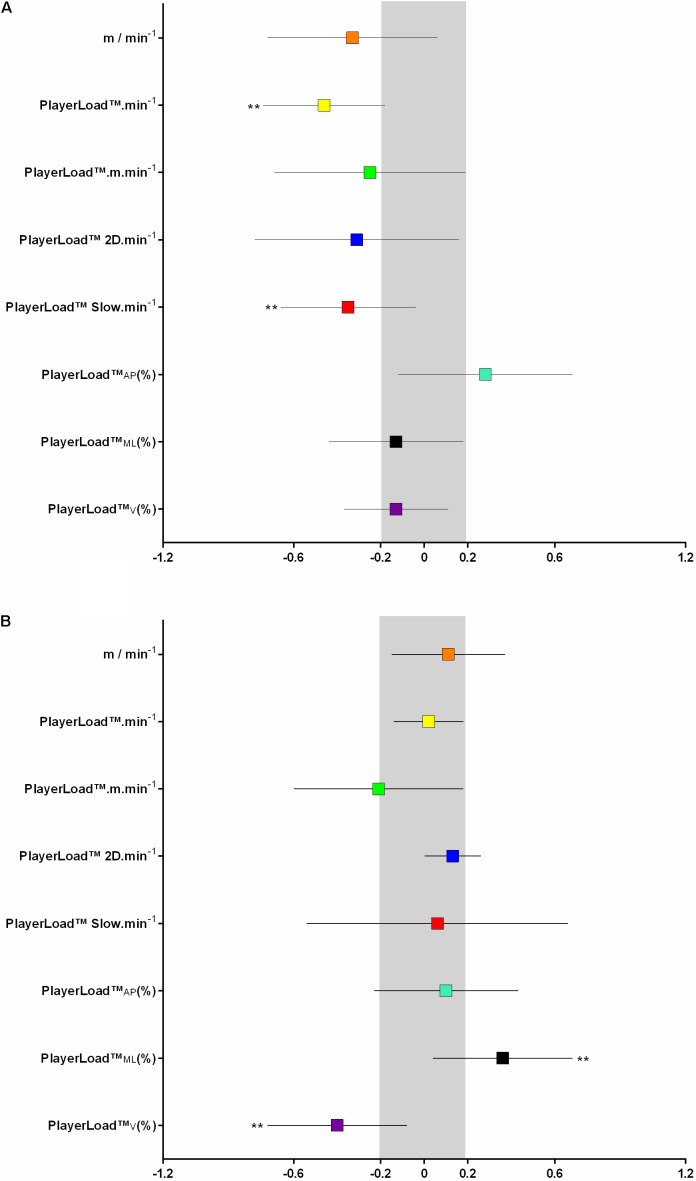
Change in small-sided game; SSG activity profile metrics **(A)** and match exercise intensity **(B)** based on flight time:contraction time; FT:CT < and > baseline by more than the 8 %CV. Symbols denote a ^∗∗^likely, ^∗∗∗^very likley, and ^∗∗∗∗^most likley chance that the change has exceeded the smallest worthwile change of 0.2 (shaded in gray).

A reduction in weekly FT:CT compared to baseline by more than 8% (CV) resulted in a *likely* reduction in SSG PlayerLoad^TM^⋅min^-1^ and PlayerLoad^TM^Slow⋅min^-1^. There were no other practically important changes (**Figure 2A**).

### Impact of FT:CT on Match Intensity

A reduction in weekly FT:CT compared to baseline by more than 8% (CV) resulted in a *likely* increase in match PlayerLoad^TM^_ML_(%) and *likely* decrease in match PlayerLoad^TM^_V_(%) (**Figure 2B**). There were no other practically important differences.

### Impact of SSG Activity Profile on Match Activity Profile

**FIGURE 3:**
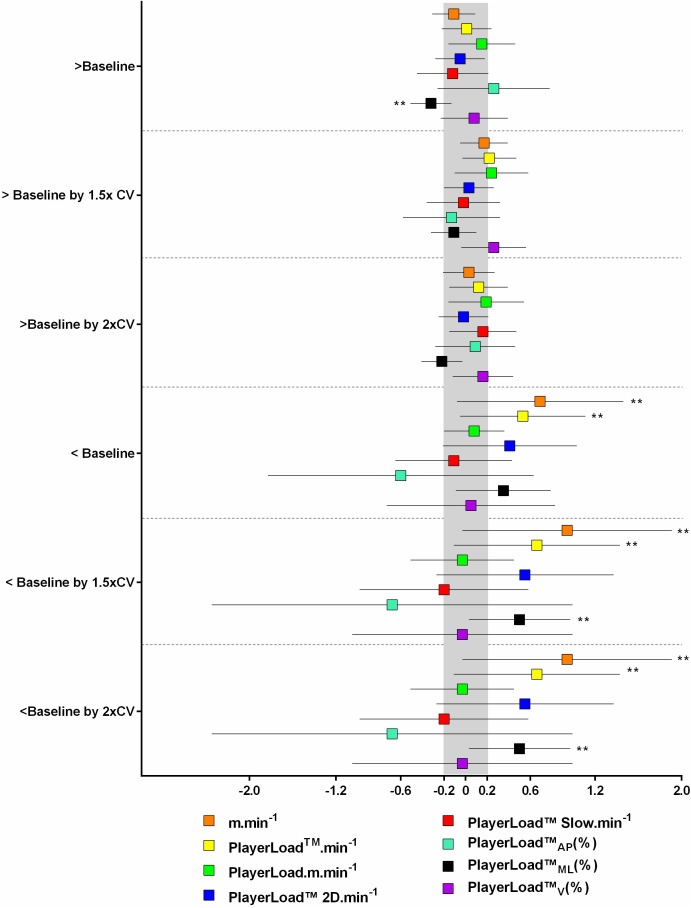
Change in match exercise intensity based on small-sided game; SSG PlayerLoad^TM^ per relative distance (meter per minute); PL⋅m⋅min^-1^ < and > baseline, by more than 1.0, 1.5, and 2.0x %CV. Symbols denote a ^∗∗^likely, ^∗∗∗^very likely, and ^∗∗∗∗^most likely chance that the change has exceeded the smallest worthwile change of 0.2 (shaded in gray).

The impact of change (as multiples of the CV%) in SSG PlayerLoad^TM^⋅m⋅min^-1^ > or < baseline by 1.0, 1.5, and 2x %CV (calculated independently for each metric) on match exercise intensity is displayed in **Figure 3**. There was a *likely* reduction in PlayerLoad^TM^_ML_(%) when SSG PlayerLoad^TM^⋅m⋅min^-1^ was greater than baseline by 1.0x the %CV. When SSG PlayerLoad^TM^⋅m⋅min^-1^ was lower than baseline, by 1.0, 1.5, and 2.0x the %CV, there was an increased match m⋅min^-1^ and PlayerLoad^TM^⋅min^-1^. Similarly there was an increase in match PlayerLoad^TM^_ML_(%) when SSG PlayerLoad^TM^⋅m⋅min^-1^ was lower than baseline by 1.5 and 2.0x %CV.

**FIGURE 4:**
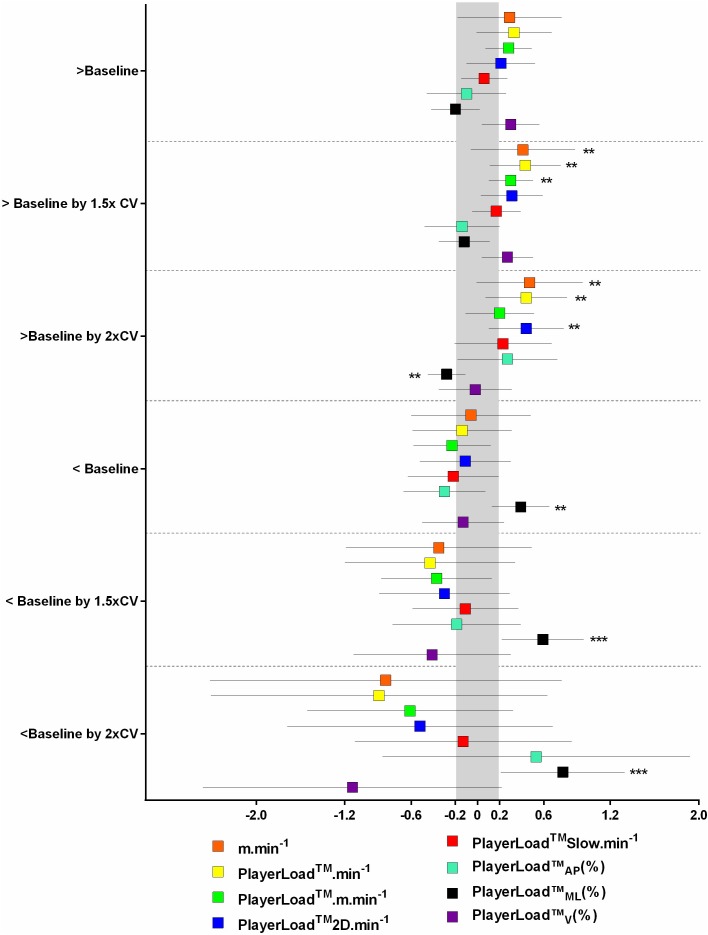
Change in match exercise intensity based on small-sided game; SSG PlayerLoad^TM^ Slow per minute; PLSlow⋅min^-1^ < and > baseline, by more than 1.0, 1.5, and 2.0x %CV. Symbols denote a ^∗∗^likely, ^∗∗∗^very likley, and ^∗∗∗∗^most likley chance that the change has exceeded the smallest worthwile change of 0.2 (shaded in gray).

**FIGURE 5:**
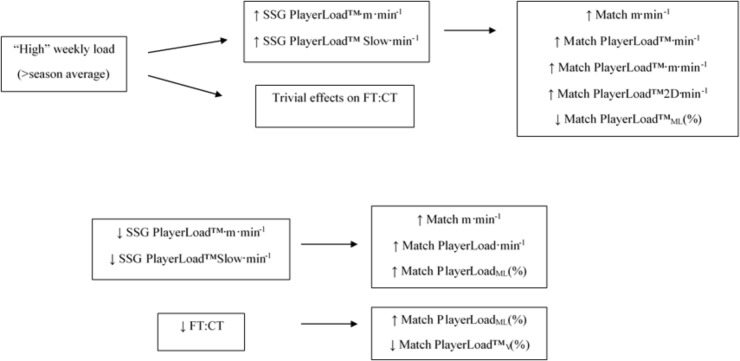
Diagrammatical representation of the impact of high weekly load compared to low weekly load, on small-sided game; SSG accelerometer derived metrics and flight time:contraction time ratio; FT:CT, the related change in match activity profile due to increased small-sided game; SSG activity profile and the impact of suppressed small-sided game; SSG activity profile and flight time:contraction time; FT:CT on subsequent match activity profile.

**Figure 4** displays the change to match exercise intensity when SSG PlayerLoad^TM^Slow⋅min^-1^ was < or > baseline by 1.0, 1.5, and 2.0x the %CV (calculated independently for each metric). When SSG PlayerLoad^TM^Slow⋅min^-1^ was greater than baseline by 1.5x the %CV, there was a *likely* higher match m⋅min^-1^, PlayerLoad^TM^⋅min^-1^ and PlayerLoad^TM^⋅m⋅min^-1^. In a similar response, an increase by 2.0x the %CV resulted in *likely* increased match m⋅min^-1^, PlayerLoad^TM^⋅min^-1^, and PlayerLoad^TM^2D⋅min^-1^, but a *likely* reduction in PlayerLoad^TM^_ML_(%). When SSG PlayerLoad^TM^Slow⋅min^-1^ was lower than baseline, there was a *likely* increase in match PlayerLoad^TM^_ML_(%). When SSG PlayerLoad^TM^Slow⋅min^-1^ was lower than baseline by 1.5 and 2.0x the %CV there was a *very likely* increase in match PlayerLoad^TM^_ML_(%). **Figure 5** summarizes the impact of load on SSG and FT:CT and subsequent impact on match exercise intensity.

## Discussion

The major finding of this study is that changes in an athlete’s activity profile during a standardized SSG (in the form of reductions in PlayerLoad^TM^⋅m⋅min^-1^ and PlayerLoad^TM^Slow⋅min^-1^) performed on a weekly basis had a direct impact on match exercise intensity via a change in movement strategy [i.e., increased contribution of PlayerLoad^TM^_ML_(%) to global PlayerLoad^TM^]. The modifications in movement strategy during a match mirror the changes seen when FT:CT is suppressed. As a result, a standardized SSG is a useful tool for the assessment of NMF.

### SSG Reliability

The reliability of speed and distance variables assessed during the SSG in this study are similar to those reported by others (Hill-Haas et al., 2008). A CV of 10% has been used as a threshold to declare a variable reliable (Duthie et al., 2003; Jennings et al., 2010). In the current study, m⋅min^-1^ was well below this value and as a result appears to be a stable metric during a SSG. However, the high CV% of HIR⋅min^-1^ suggests there may be limited scope for the use of this variable in examining SSG activity profile. Furthermore, the CV% of the accelerometer variables analyzed in this study are comparable to those determined from treadmill running, Australian Rules Football training and matchplay, and rugby matchplay (Boyd et al., 2013; Barrett et al., 2014; Kempton et al., 2015; McLaren et al., 2016). Given PlayerLoad^TM^ is likely to represent a more global measure of external load (Boyd et al., 2013; Barrett et al., 2014; Cormack et al., 2014) the results of this study suggest that PlayerLoad^TM^ and its variants have the potential to be sensitive indicators of modifications in SSG activity profile.

### Impact of Weekly Load on SSG Activity Profile and FT:CT

The first finding from this work is that relatively high preceding weekly training load results in an increase in accelerometer based metrics (i.e., PlayerLoad^TM^⋅m⋅min^-1^, PlayerLoad^TM^Slow⋅min^-1^) during a standardized SSG. These results are somewhat similar to the within-match changes described by Barrett et al. (2016a). In this work, the authors speculated that the increase in PlayerLoad^TM^⋅m⋅min^-1^ seen late in the match was representative of decreased efficiency of movement due to match induced fatigue (Barrett et al., 2016a). In isolation, if an increase in PlayerLoad^TM^⋅m⋅min^-1^ is in fact a reflection of a fatigue induced change in movement strategy, the results found here might suggest that a high weekly load has a similar fatigue effect on movement efficiency as brought about by match play. These changes may reflect a reduction in stride length, resulting in more frequent strides and foot contacts or movements at a lower velocity (Cormack et al., 2013; Barrett et al., 2016a). Critically, however, the work of Barrett et al. (2016a) did not measure fatigue *per se*, to allow a comparison with the increase in PlayerLoad^TM^⋅m⋅min^-1^ detected. The increase in PlayerLoad^TM^⋅m⋅min^-1^ could in fact represent an increase in the amount of acceleration, deceleration, and change of direction relative to distance covered (Dalen et al., 2016). This may be indicative of an “end spurt” resulting in an increase in activity profile rather than a manifestation of fatigue during the latter stages of the match (Bradley and Noakes, 2013; Paul et al., 2015). Furthermore, the use of global PlayerLoad^TM^ rather than the contribution of the individual vectors provides limited information on specific changes in movement strategy that might occur in fatigue (Cormack et al., 2013; Mooney et al., 2013; Dalen et al., 2016).

Another potential explanation for the increase in PlayerLoad^TM^⋅m⋅min^-1^ during the SSG seen following high weekly training load, could be that as players are aware they have a match to play the following day, they modify their SSG movements as a kind of “pacing strategy” in response to perceived fatigue (Krustrup et al., 2002; Weston et al., 2011). Previous work has shown that perceived wellness impacts subsequent external training load and a similar mechanism may be at play here (Gallo et al., 2016). Despite these possibilities, an important factor with the current results could be that although “high” load weeks in this study are a sufficient stimulus to modify SSG activity profile seen as an increase in PlayerLoad^TM^⋅m⋅min^-1^ and a metric that has previously reflected low velocity exertions; PlayerLoad^TM^Slow⋅min^-1^ (McLaren et al., 2016), these modifications are in fact not manifestations of NMF (see below for further discussion of this concept).

Whilst it was shown that a relatively high weekly load resulted in changes to SSG activity profile, the same load did not result in substantial changes to FT:CT. This variable has been demonstrated to be an ecologically valid representation of NMF in both Australian Rules Football and A-League Football (Cormack et al., 2008a, 2013; Rowell et al., 2016). Importantly, this variable has displayed a dose-response relationship to match play in A-League players and an association with performance in Australian Rules Football players (Cormack et al., 2008b; Mooney et al., 2013; Rowell et al., 2016). Given these previous findings, it could reasonably be expected that if the players in this study were in fact in a state of NMF due to high preceding load, that this variable would have detected it. Therefore, as players did not show meaningful reductions in FT:CT due to high training load, it appears that the changes seen in SSG activity profile due to high load may in fact not represent a negative (i.e., fatigue) response to previous training. This suggestion is supported by indicating a change in movement strategy (represented by a reduction in the contribution of the vertical vector to PlayerLoad^TM^⋅min^-1^) during Australian Rules Football matches when players were experiencing NMF (Cormack et al., 2013; Mooney et al., 2013). As mentioned above, although the increase in PlayerLoad^TM^⋅m⋅min^-1^ has been suggested as indicative of match induced fatigue (Barrett et al., 2016a), the changes in accelerometer variables seen here may actually be a positive adaptive response to the previous high load (Aughey et al., 2016). Given the trivial changes seen here in FT:CT, it appears that the “high load” weeks as defined in this work were not a clear cause of NMF. Furthermore, the ability in this study to assess changes in SSG activity profile changes in parallel to changes in FT:CT suggests that increases in PlayerLoad^TM^⋅m⋅min^-1^ during matches, previously proposed as representing fatigue (Barrett et al., 2016a), are questionable.

### Impact of FT:CT on SSG Activity Profile

When FT:CT was reduced, there were concomitant reductions in PlayerLoad^TM^⋅min^-1^ and PlayerLoad^TM^Slow⋅min^-1^ during the SSG. As suggested, it appears that those weeks classified as “high” (relative to each players own season average) in the current study did not in isolation represent a sufficiently fatiguing stimulus to suppress FT:CT. This may be due to the fact that a preceding match has been shown to disturb this variable whereas training typically does not, presumably due to the lower volume and/or intensity of training sessions compared to matches (Cormack et al., 2008a,b; Rowell et al., 2016). Given that FT:CT typically recovers in A-League footballers by 42 h post-match (Rowell et al., 2016), it appears that in this case, the timing of collection of FT:CT since the previous match, has meant that players have generally returned to baseline (Rowell et al., 2016). However, in situations where FT:CT remains suppressed, SSG activity profile on the same day as FT:CT has also been altered. This provides an indication that SSG activity profile is sensitive to NMF. The changes seen are somewhat similar to the modifications to movement strategy seen during matches in Australian Rules Footballers who have a reduced FT:CT (Cormack et al., 2013), although the underlying mechanism responsible for these alterations is unable to be determined from the current data.

### Impact of FT:CT on Match Intensity

Although the impact of preceding load on the changes in activity profile of a standardized SSG and FT:CT may be of interest, the key to the usefulness of a protocol as an ongoing monitoring tools lies in its ability to reflect changes in subsequent match exercise intensity and/or performance (Cormack et al., 2013; Mooney et al., 2013). In other words, whilst a particular protocol may change relative to preceding load (i.e., match and/or training), if this change has no impact on exercise intensity, movement strategy, or performance within a match played in close proximity to the protocol, then the relevance of any changes seen are somewhat questionable. Similar to the impact on SSG activity profile seen with a reduced FT:CT, a suppression of FT:CT also elicited an alteration in match exercise intensity in the form of increased PlayerLoad^TM^_ML_(%) and lower PlayerLoad^TM^_V_(%). The reduction in the contribution of the vertical vector to PlayerLoad^TM^ is identical to previous results in Australian Rules Football players that were classified as fatigued (Cormack et al., 2013). There are numerous potential mechanisms that might explain this finding. As suggested previously, reduced FT:CT might represent NMF that directly limits the ability for high intensity movements and this may be related to a reduction in vertical stiffness (Girard et al., 2011; Cormack et al., 2013; Gaudino et al., 2013; Mooney et al., 2013; Buchheit et al., 2015). Alternatively, a reduction in FT:CT could result in an increased perception of effort required in order to produce a given external load, causing a reduction in high intensity movements (and resultant contribution of the vertical vector to total PlayerLoad^TM^) (Cormack et al., 2013; Blanchfield et al., 2014). Furthermore, reductions in perceived wellness from a subjective questionnaire have been demonstrated to impact the relationship between perception of effort and external load measured via PlayerLoad^TM^ and similar mechanisms may be involved here (Gallo et al., 2015).

### Impact of SSG Activity Profile on Match Exercise Intensity

Whilst the impact of reductions to FT:CT on subsequent match exercise intensity have previously been investigated (Mooney et al., 2013) and also shown to be important in the current study, to our knowledge no study has examined the impact of changes in SSG activity profile (i.e., as a representation of NMF) on match exercise intensity. In the current study, an increase in SSG PlayerLoad^TM^⋅m⋅min^-1^ above baseline resulted in a reduction to PlayerLoad^TM^_ML_(%) and increase in PlayerLoad^TM^_V_(%) during a match. The increase in PlayerLoad^TM^_V_(%) during the match as result of elevated SSG PlayerLoad^TM^⋅m⋅min^-1^ is opposite to the reduction in the contribution of the vertical vector to PlayerLoad^TM^ that has been shown in Australian Rules Football players competing in a state of NMF (Cormack et al., 2013). This adds further weight to the suggestion that an increase in PlayerLoad^TM^⋅m⋅min^-1^ during the SSG (or other competitive situations) is in fact not a representation of a fatigue state.

However, in contrast, when SSG PlayerLoad^TM^⋅m⋅min^-1^ and PlayerLoad^TM^Slow⋅min^-1^ were lower than baseline, a similar pattern in match intensity to that of a reduced FT:CT was evident. That is, an increase in PlayerLoad^TM^_ML_(%) during the match and whilst unclear (suggesting individual variability) a potential reduction in PlayerLoad^TM^_V_(%). This finding provides additional support to the notion that “high load” as defined in this study does not represent a negative (i.e., fatiguing stimulus). The “high loads” in this study appear to be a positive stimulus and is similar to previous work suggesting improved match performance from relatively higher training load (Aughey et al., 2016; Gabbett, 2016).

The results of this study suggest that reductions in specific SSG activity profile variables coincide with changes to match exercise intensity in the form of altered movement strategy. Specifically, the substantial reduction (greater than the %CV) in weekly SSG PlayerLoad^TM^⋅m⋅min^-1^, compared to baseline, resulted in a *likely* increase in match m⋅min^-1^, PlayerLoad^TM^⋅min^-1^ and PlayerLoad^TM^_ML_(%). Similarly a substantial reduction in weekly SSG PlayerLoad^TM^Slow⋅min^-1^ compared to baseline displayed a *likely to very likely* increase in match PlayerLoad^TM^_ML_(%). Critically, the resultant changes in match exercise intensity are similar to those evident when FT:CT is reduced [i.e., *likely* increase in match PlayerLoad^TM^_ML_(%) also observed when reduced SSG PlayerLoad^TM^⋅m⋅min^-1^ and PlayerLoad^TM^Slow⋅min^-1^]. The reliability and ecological validity of FT:CT has previously been established (Cormack et al., 2008a,b; Rowell et al., 2016), however, the changes in match exercise intensity shown here add further weight to the value of this metric for assessing NMF in field sport athletes. As mentioned previously, there are numerous potential mechanisms that may explain this outcome, although they are somewhat speculative as they have not been specifically measured in the current study (Girard et al., 2011; Cormack et al., 2013; Gaudino et al., 2013; Mooney et al., 2013; Buchheit et al., 2015).

A novel outcome of this work is that accelerometer based metrics (PlayerLoad^TM^⋅m⋅min^-1^ and PlayerLoad^TM^Slow⋅min^-1^) collected from the performance of a standardized SSG appear to be a useful surrogate for a test of NMF such as FT:CT. Due to the time constraints and potential equipment limitations of performing a CMJ to assess FT:CT, coaches and practitioners may be able to determine similar information from a SSG performed as part of a warm up or during an on-field training session. Previous work has suggested the potential for the use of standardized protocols to collect heart rate recovery and heart rate variability data in the field (Buchheit et al., 2008, 2010, 2012). Although potentially valuable, this approach is somewhat limited as heart rate data needs to be collected in a stand-alone protocol. The results of this study suggest that a standardized SSG may simultaneously be able to deliver physiological, technical, tactical and fatigue assessment outcomes. It should be acknowledged, however, that a higher medio-lateral contribution to PlayerLoad^TM^ has been observed when the accelerometer is placed at the centre of mass and higher vertical contribution when positioned at the scapula (Barrett et al., 2016b). As players in this study always wore units between the scapula in a custom tight fitting vest, the changes in vector contributions seen here are likely to represent meaningful modifications.

## Conclusion and Practical Applications

From a practical perspective, reductions in PlayerLoad^TM^⋅m⋅min^-1^ and PlayerLoad^TM^Slow⋅min^-1^ during a standardized SSG appear to have the same implications for match exercise intensity (i.e., movement strategy) as reduction in FT:CT [i.e., increased contribution of PlayerLoad^TM^_ML_(%) to global PlayerLoad^TM^]. There is also the potential that given the individual variability shown here, that if PlayerLoad^TM^Slow⋅min^-1^ is lower in a pre-match SSG compared to baseline, reduced PlayerLoad^TM^_V_(%) may present in match play providing it is assessed on an individual level. Practitioners may consider the use of a regular standardized SSG for the assessment of NMF. Future work should examine in more detail the impact of match performance in the presence of NMF detected via a SSG.

## Author Contributions

AR, SC, RA, and JC: conceived and designed the study, drafted the manuscript, prepared the tables and figures, edited and critically revised the manuscript, and approved the final version. AR: data collection and analysis. AR and SC: statistical data analysis and interpretation. AR, SC, and RA: interpreted the results.

## Conflict of Interest Statement

The authors declare that the research was conducted in the absence of any commercial or financial relationships that could be construed as a potential conflict of interest.
